# SmartGel OV: A Natural *Origanum vulgare*-Based Adjunct for Periodontitis with Clinical and Microbiological Evaluation

**DOI:** 10.3390/medicina61081423

**Published:** 2025-08-07

**Authors:** Casandra-Maria Radu, Carmen Corina Radu, Dana Carmen Zaha

**Affiliations:** 1 Doctoral School of Biological and Biomedical Sciences, University of Oradea, 1 University Street, 410087 Oradea, Romania; rcasandra1996@gmail.com (C.-M.R.);; 2 Department of Forensic Medicine, George Emil Palade University of Medicine, Pharmacy, Science, and Technology of Targu Mures, 38 Gheorghe Marinescu Street, 540139 Targu Mures, Romania; 3 Department of Preclinical Disciplines, Faculty of Medicine and Pharmacy, University of Oradea, 1 December Square, 410028 Oradea, Romania

**Keywords:** real-time PCR, periodontitis, SmartGel OV, AI diagnostics

## Abstract

*Background and Objectives*: Periodontitis is a chronic inflammatory disease that leads to progressive destruction of periodontal tissues and remains a significant global health burden. While conventional therapies such as scaling and root planning offer short-term improvements, they often fall short in maintaining long-term microbial control, underscoring the need for adjunctive strategies. This study evaluated the clinical and microbiological effects of a novel essential oil (EO)-based gel—SmartGel OV—formulated with *Origanum vulgare*. *Materials and Methods*: Thirty adults with periodontitis were enrolled in a 4-month observational study, during which SmartGel OV was applied daily via gingival massage. Clinical outcomes and bacterial profiles were assessed through probing measurements and real-time PCR analysis. Additionally, a pilot AI-based tool was explored as a supplemental method to monitor inflammation progression through intraoral images. *Results*: Significant reductions were observed in *Fusobacterium nucleatum* and *Capnocytophaga* spp., accompanied by improvements in clinical markers, including probing depth, bleeding on probing, and plaque index. The AI framework successfully identified visual inflammation changes and supported early detection of non-responsiveness. *Conclusions*: SmartGel OV demonstrates promise as a natural adjunctive treatment for periodontitis and AI monitoring was included as an exploratory secondary tool to assess feasibility for future remote tracking.

## 1. Introduction

The oral microbiome is a diverse ecosystem of microorganisms, including bacteria, viruses, fungi, and other microbes, that inhabit the oral cavity. These microbes thrive on various surfaces, including teeth, gums, cheeks, and the throat, forming a complex and dynamic system essential for maintaining oral health and influencing overall well-being [1,2,3]. This ecosystem is continually exposed to physical and chemical disruptions, making its balance dependent on individual habits and anatomical factors. When the balance of commensal microorganisms is disrupted, pathogenic bacteria can dominate, leading to oral health issues such as dental caries, oral cancer, and other diseases [4,5,6].

Periodontal disease, one of the most common oral conditions, affects the supporting soft and hard tissues of the teeth, encompassing gingivitis and periodontitis. Gingivitis arises from the accumulation of bacterial plaque near the gingival sulcus, resulting in symptoms such as bleeding, swollen gums, and discomfort [7,8].

Periodontitis is a multifactorial, biofilm-associated chronic inflammatory disease affecting tooth-supporting structures, including the cementum, periodontal ligament (PDL), alveolar bone, and gingival tissue. If untreated, it can result in the loss of both soft and hard tissue [9,10]. This condition is characterized by clinical loss of attachment, radiographic evidence of bone resorption (both vertical and horizontal), the formation of periodontal pockets, and bleeding upon palpation, making it a globally prevalent issue [11,12,13].

Beyond its implications as a dental disease, chronic periodontitis has gained significant attention due to its systemic impact. It is associated with unresolved hyperinflammation; disruptions in the adaptive immune system; and dysbiosis of microbiota in the oral cavity, gut, and other locations. These systemic effects may contribute to, coexist with, or exacerbate other health conditions, increasing morbidity [14,15].

Periodontitis has been linked to numerous systemic diseases, including cardiovascular diseases, type 2 diabetes mellitus, respiratory conditions, chronic kidney disease (particularly in dialysis patients), metabolic syndrome, Alzheimer’s disease, preterm birth, and even lung cancer [16,17]. According to the WHO Global Oral Health Status Report (2022) [18], approximately 19% of the global adult population suffers from severe periodontitis, amounting to over 1 billion cases worldwide—a significant increase compared to the findings of Kassebaum et al. (2014) [19].

Periodontitis is a leading cause of edentulism and one of the most severe oral pathologies due to its role in activating osteoclastogenesis, which leads to alveolar bone destruction. Over time, the disease often progresses with minimal or no noticeable symptoms, leaving it undetected or unrecognized by patients. Despite being relatively simple and cost-effective to treat in its early stages, periodontitis imposes a substantial global economic burden, costing billions of dollars annually. Therefore, early diagnosis and adjunctive therapies that enhance the efficacy of conventional mechanical debridement are essential for improving outcomes [4,20].

Chronic inflammation arises from microbial infections within the subgingival biofilm, primarily driven by periodontal pathogens collectively known as the “red complex” (*Porphyromonas gingivalis*, *Treponema denticola*, and *Tannerella forsythia*) [21]. These Gram-negative anaerobes are strongly linked to the onset and progression of periodontal disease. They play a crucial role in the disease process by initiating and sustaining the chronic inflammatory response that leads to tissue destruction. Studies have shown that EO products can effectively reduce these bacteria and biofilm formation in certain cases [8,22,23].

Despite the widespread use of non-surgical interventions such as scaling and root planning (SRP), residual inflammation and microbial recolonization often persist in deeper pockets, furcation areas, and anatomically complex sites. Therefore, recent clinical guidelines advocate for adjunctive therapeutic approaches that enhance the efficacy and longevity of mechanical debridement [24,25,26]. These adjuncts may include antibiotics, antiseptics, probiotics, and more recently, plant-derived natural products with therapeutic potential.

Among plant-based therapies, essential oils (EOs) have demonstrated promising antimicrobial, anti-inflammatory, and antioxidant properties [27,28]. One EO of particular interest in periodontitis research is *Origanum vulgare* (oregano) EO, rich in phenolic monoterpenes such as carvacrol and thymol, which exhibits strong inhibitory effects against major periodontal pathogens and modulates host inflammatory responses [29,30]. However, clinical evidence supporting its use in human periodontitis remains limited, and further investigation is required [31,32,33].

The present study aimed to evaluate the clinical and microbiological effects of SmartGel OV, a topical gel containing *Origanum vulgare* EO, as an adjunctive therapy in patients with periodontitis. Specifically, we sought to determine its impact on key clinical parameters (probing depth, clinical attachment level, bleeding on probing, and plaque index) and on the reduction of major periodontal pathogens. Additionally, we explored the potential of an AI-based inflammation scoring tool as a secondary objective.

## 2. Materials and Methods

### 2.1. Study Design and Participants

This observational cohort study included 30 adult patients (17 females, 13 males; age range: 32–60 years) diagnosed with periodontitis. Participants were recruited from a private dental clinic in Oradea, Romania, between June and July 2024. Inclusion criteria were: (1) moderate to severe periodontitis, (2) no antibiotic use in the prior 3 months, (3) absence of systemic diseases, and (4) willingness to adhere to the study protocol for 4 months. All patients signed informed consent forms.

### 2.2. Clinical Examination and Imaging

**Figure 1:**
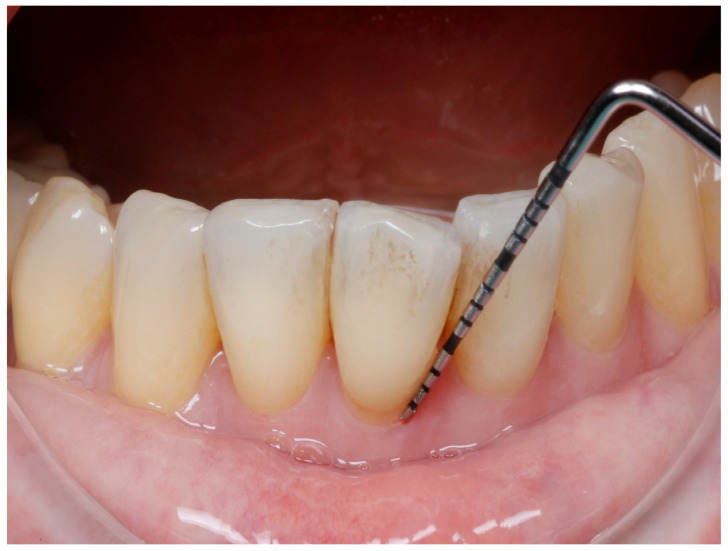
Probing depth in one vestibular site.

**Figure 2:**
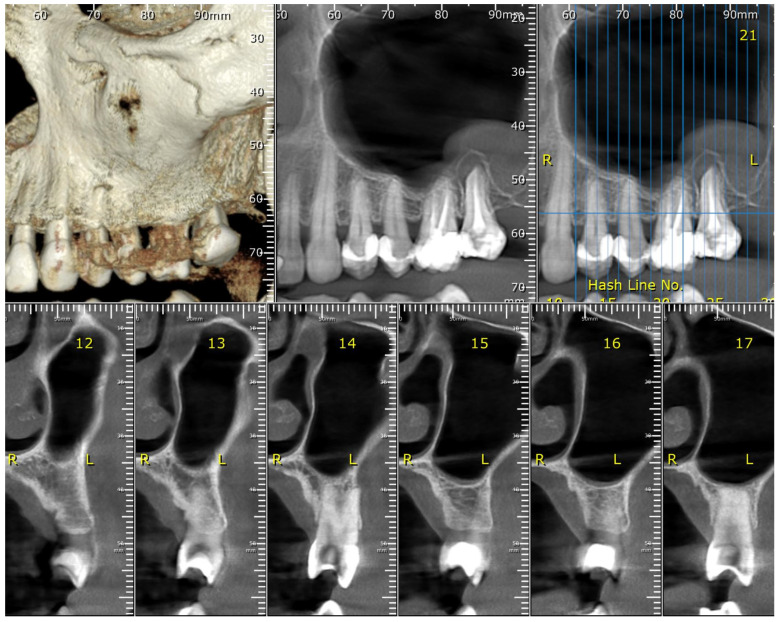
The maxillary CBCT scan reveals levels of bone loss, both vertical and horizontal.

**Figure 3:**
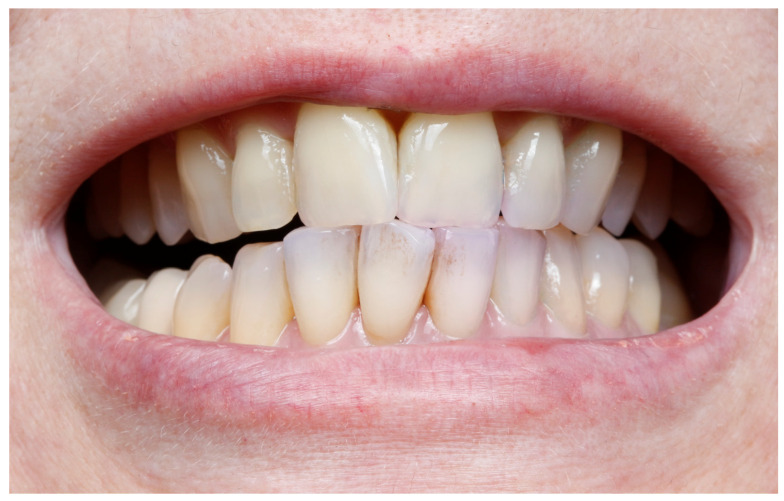
The patient is showing good hygiene. After using a colored plaque revealer, only the incisal parts of the teeth are slightly colored.

At baseline and 4-month follow-up, periodontal parameters, including PD, BOP, and CAL, were assessed using a Williams probe, as seen in Figure 1. Radiographs and intraoral photographs were taken to assess bone level and soft tissue status, as shown in Figure 2. A plaque disclosure agent was used to evaluate oral hygiene, as shown in Figure 3.

### 2.3. Intervention Protocol

All patients received a single session of professional scaling and polishing at baseline. No further mechanical therapy was provided during the study period. Twice daily, participants used SmartGel OV—a topical gel formulation based on *Origanum vulgare* EO—with anti-inflammatory and antimicrobial properties. The product was applied by gently rubbing it along the gingival margins twice a day for 4 months. In addition, SmartGel OV’s AI-powered inflammation tracking function was used once daily as part of the same application process prior to digital monitoring.

### 2.4. SmartGel OV Digital Monitoring

#### 2.4.1. Application Protocol

SmartGel OV was applied once daily by patients after routine oral hygiene. A small quantity of the gel was gently massaged along the gingival margins using a fingertip or silicone applicator. This ensured direct contact with inflamed tissues and facilitated the absorption. SmartGel OV is a topical formulation developed by PlusFarma (Oradea, Romania), composed primarily of *Origanum vulgare* EO with the following standardized ingredients, certified for cosmetic and dental use within EU compliance standards:

- Active ingredient: *Origanum vulgare* EO (2.5%)
- Base: Carbomer 940
- Humectant: Glycerin
- Preservative: Sodium benzoate
- pH Adjuster: Triethanolamine
- Solvent: Purified water

#### 2.4.2. Exploratory Digital Monitoring Using AI

As an exploratory component, a conventional neural network (CNN)-based inflammation scoring model was used. The CNN model, trained on over 3000 annotated periodontal image samples, detected features such as gingival redness, swelling, marginal recession, and suspected BOP. To ensure model generalizability and robustness, we employed a stratified 5-fold cross-validation approach during training. This method allowed each subset of the data to serve as a validation set once, while the remaining four-fifths were used for training, minimizing overfitting and improving external reliability.

Model performance was evaluated on a hold-out test set (20% of the total data). The CNN achieved an overall accuracy of 91.3%, with a sensitivity of 88.6% and specificity of 93.9%, indicating a strong discriminatory capability for detecting gingival inflammation [34,35].

The system generated the following outputs:

1. A numeric inflammation severity score (scale 0–3),
2. Heatmap overlays indicating localized inflammation zones,
3. Identification of asymmetrical gingival inflammation,
4. Microbial trend projections based on previous GCF data,
5. AI-based prediction of SmartGel OV responsiveness.

A secure clinician dashboard allowed for real-time monitoring of each patient’s inflammation progression. This semi-automated model enabled remote follow-up and improved patient engagement through personalized feedback loops. This AI system was not part of the primary analysis and served as an adjunct to explore feasibility for future applications in remote patient monitoring.

##### Illustrative Example: AI Monitoring Workflow

A 44-year-old female patient with moderate periodontitis uploaded images at designated intervals.

- Baseline: Received an AI-calculated inflammation score of 2.3, with heatmaps indicating high redness and asymmetry in the upper right quadrant.
- Week 2: Inflammation score dropped to 1.7; AI confirmed early therapeutic response.
- Month 2: Score reduced to 1.0 with microbial reduction confirmed by PCR.
- Month 4: Final score reached 0.3, aligned with complete clinical recovery.

**Figure 4:**
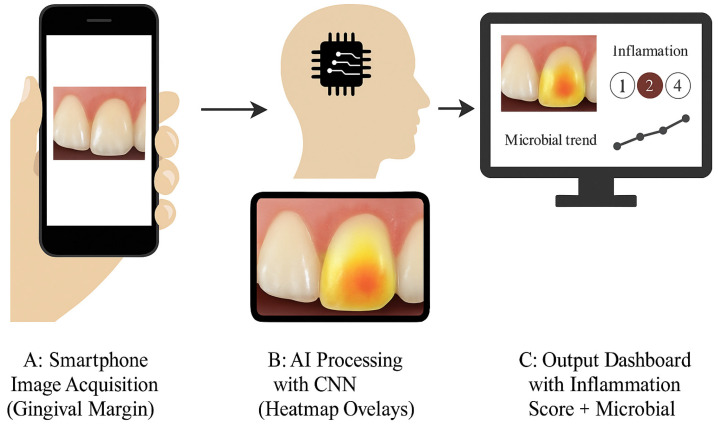
AI-supported periodontal monitoring framework. Panel (**A**): The smartphone captures an intraoral image of the gingival margin. Panel (**B**): AI heatmap overlay of inflammation zones. Panel (**C**): Output dashboard showing inflammation severity score and microbial trends.

This case illustrates how SmartGel OV’s AI companion provided predictive insights, enabling clinicians to tailor care dynamically, as shown in Figure 4.

### 2.5. Microbial Sampling and Analysis

**Figure 5:**
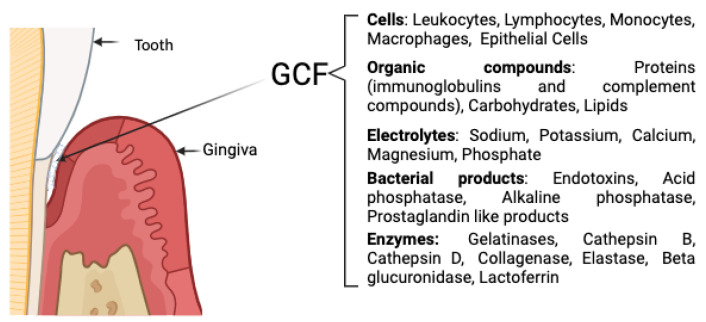
Composition of GCF [33,38,39].

**Figure 6:**
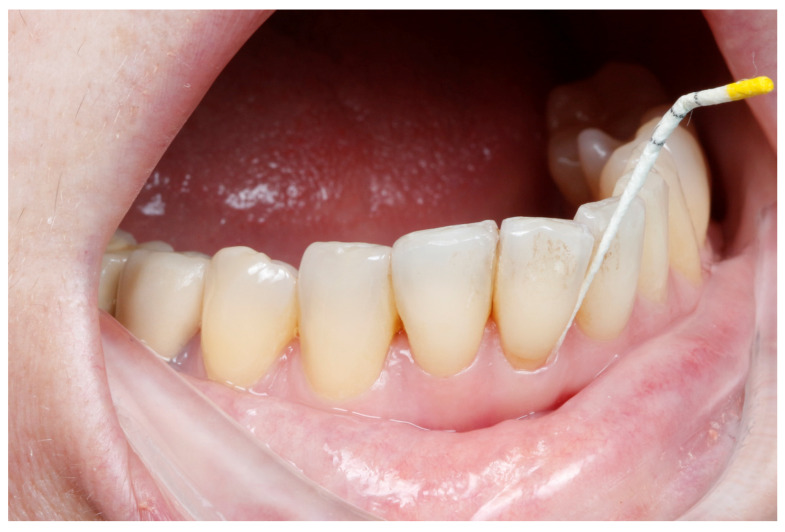
GCF sample collection using a paper point.

GCF, as shown in Figure 5, was collected from five preselected periodontal pockets per patient using ISO 30 filter paper points (IMD Labor, Berlin, Germany) [36]. Sites were isolated with cotton rolls and air-dried before sampling. Samples were collected 2–3 h postprandially and stored at 4 °C in phosphate-buffered saline, as shown in Figure 6. Only blood-free samples were used [37].

PCR analysis was conducted at a certified microbiology laboratory to quantify 11 major periodontopathic bacteria: *Actinobacillus actinomycetemcomitans* (Aa), *Prevotella intermedia* (Pi), *Porphyromonas gingivalis* (Pg), *Tannerella forsythia* (Tf), *Treponema denticola* (Td), *Parvimonas micra* (Pm), *Fusobacterium nucleatum*/*periodonticum* (Fn), *Campylobacter rectus* (Cr), *Eubacterium nodatum* (En), *Eikenella corrodens* (Ec), and *Capnocytophaga* spp. (Cp).

Samples were eluted overnight at 4 °C and centrifuged at 400× *g* for 5 min before DNA extraction. Total genomic DNA was isolated using a silica-column-based protocol and analyzed via real-time quantitative PCR (qPCR) on a QuantStudio™ 5 Real-Time PCR System (Thermo Fisher Scientific, Waltham, MA, USA), utilizing SYBR Green detection chemistry. Each reaction consisted of 25 μL containing master mix, species-specific primers (0.3 μM), and DNA template. Representative primer sequences used included:

- P. gingivalis: F 5′-AGGCAGCTTGCCATACTGCG-3′/R 5′-ACTGTTAGCAACTACCGATGT-3′
- T. forsythia: F 5′-AGAGTGCTTCTTCGTTGACT-3′/R 5′-TAAGGCGGTCGCTAGTAGG-3′

Thermocycling conditions included an initial denaturation at 95 °C for 3 min, followed by 40 cycles of 95 °C for 15 s and 60 °C for 30 s. Melting curve analysis was performed at the end of each run to ensure amplicon specificity. Positive and no-template controls were included in every PCR run. A Ct value of <38 was defined as positive according to the laboratory’s validation criteria.

### 2.6. Ethical Considerations

The local ethics committee approved this study. All patients were informed about the purpose, procedures, and potential risks before enrolment.

### 2.7. Statistical Analysis

All data were analyzed using DataLab (DATAtab Team (2023). DATAtab: Online Statistics Calculator. DATAtab e.U. Graz, Austria. URL https://datatab.net (accessed on 10 July 2024)). Descriptive statistics were used to summarize the clinical parameters and microbial prevalence. Continuous variables such as PD, CAL, BOP, and PI were expressed as mean ± standard deviation (SD). Paired two-tailed *t*-tests were used to compare baseline and 4-month values. A *p*-value < 0.05 was considered statistically significant.

Microbial prevalence rates before and after treatment were compared using the McNemar test for paired nominal data.

## 3. Results

Of the 30 enrolled patients, 28 completed the full 4-month protocol. Two participants withdrew due to personal scheduling constraints unrelated to the study. No adverse effects were reported from the use of SmartGel OV. All clinical parameters (PD, CAL, BOP, and PI) demonstrated statistically significant improvement after 4 months of SmartGel OV application (*p* < 0.05). The reduction in microbial prevalence, particularly for *F. nucleatum*, *Pg*, and *Cp*, was also statistically significant, as determined by the McNemar test. Full results are summarized in Table 1 and Table 2.

### 3.1. Clinical Outcomes

Periodontal clinical parameters showed consistent improvement across the cohort. Table 1 summarizes the average values recorded at baseline and the 4-month follow-up. The improvements were statistically significant (*p* < 0.05) in all measured parameters.

### 3.2. Microbiological Findings

GCF samples were successfully collected and analyzed in all 28 patients. At baseline, 82% of patients demonstrated colonization by at least one pathogen from the red complex or orange complex. After 4 months of SmartGel OV application, the microbial load was reduced across all species tested. Notably, *Fusobacterium nucleatum* and *Capnocytophaga* spp. were present in 42.9% and 35.7% of patients, respectively, at baseline, but dropped to 10.7% and 7.1% post-treatment, respectively.

### 3.3. AI-Based Inflammation Monitoring

The AI component of SmartGel OV successfully tracked the progression of inflammation via monthly intraoral images. At baseline, the average AI-generated inflammation score was 2.3 (on a scale of 0–3). By the end of the study, the average score had dropped to 0.6.

Heatmap overlays indicated early response in the first two weeks, which was predictive of clinical improvement at 4 months. Patients who showed slower visual improvement, as analyzed by AI, also had higher residual bacterial counts, indicating good agreement between the imaging and molecular data.

The system also detected asymmetrical inflammation in 46% of cases, prompting localized reapplication or follow-up by the clinical team.

These findings suggest that SmartGel OV not only reduced microbial and clinical signs of periodontitis, but also enabled dynamic, AI-driven inflammation tracking to support proactive care.

## 4. Discussion

The present study evaluated the therapeutic efficacy of SmartGel OV, a gel containing *Origanum vulgare* EO, used as an adjunctive treatment for periodontitis. Over a four-month period, its application resulted in notable improvements in clinical parameters (PD, CAL, BOP, and PI), significant reductions in key periodontopathogens, and a parallel decrease in inflammation scores tracked using a novel CNN-based AI system.

These results align with emerging evidence supporting the use of EO-based formulations and AI-guided monitoring tools in periodontal therapy. One of the key strengths of this study is the integration of natural adjunctive therapy with digital inflammation tracking, addressing two persistent challenges in periodontitis management: the need for non-invasive, sustained antimicrobial support and improved adherence monitoring. Traditional mechanical treatments, while effective in the early stages, often fail to maintain long-term microbial control in deep pockets or complex cases. By incorporating an EO with both antimicrobial and anti-inflammatory potential, SmartGel OV offers a targeted adjunct that can be applied locally and consistently over extended periods. This is particularly important in chronic cases where microbial recolonization and low-grade inflammation often persist.

Mechanistically, *Origanum vulgare* EO exhibits a dual mode of action. First, its high content of carvacrol and thymol exerts bacterial effects by disrupting the lipid bilayer of bacterial membranes, and inhibiting ATP synthesis. In our study, the most significant reductions were observed in *F. nucleatum* (from 42.9% to 10.7%) and *Capnocytophaga* spp. (from 35.7% to 7.1%), species known for their role as bridging organisms in microbial successions. The reduction of *P. gingivalis* and *T. forsythia*—both part of the red complex—further underscores the antibacterial effectiveness of the gel.

The anti-inflammatory properties of *O. vulgare* are mediated through the modulation of host immune responses. In our study, this effect was indirectly validated through the AI-monitored decrease in inflammation severity scores. Patients experienced a rapid drop in inflammation scores within the first two weeks, often preceding the measurable reduction in microbial load. This suggests that the gel may help interrupt the feedback loop between microbial persistence and host-driven tissue destruction.

The gel’s formulation components may also contribute to its synergistic effects. Glycerin functions as humectant and improves mucosal hydration, potentially enhancing the retention and bioavailability of active compounds. The carbomer base ensures controlled viscosity and spreadability, allowing for better surface coverage of the gingival margin. These formulation features, combined with the EO’s active ingredients, likely created a favorable local environment for tissue recovery and microbial suppression.

While not central to the study’s primary aim, a CNN-based model was piloted to evaluate inflammation changes via intraoral photographs, providing clinicians with numeric inflammation scores, heatmap overlays, and predictive alerts. Importantly, our CNN model achieved strong performance metrics (AUC = 0.948; sensitivity = 88.6%; specificity = 93.9%), and its outputs showed strong concordance with GCF PCR trends. This suggests that AI-guided monitoring could serve as a surrogate for traditional clinical follow-ups, especially in underserved or remote populations. Prior studies have demonstrated the utility of AI in detecting caries and oral lesions; however, its integration into dynamic periodontal tracking remains a novel approach and this component should be interpreted as preliminary and exploratory.

Nonetheless, this study has limitations. First, the observational nature and absence of a control group prevent definitive attribution of results to SmartGel OV alone. Second, the sample size (*n* = 30) was limited and drawn from a single geographic area, reducing generalizability. Third, while PCR provides high specificity for microbial shifts, it does not assess total microbial diversity or functional activity. Fourth, adherence to both the gel and image submission protocol was self-reported, introducing a potential for bias. Finally, the study’s four-month duration does not provide information on the long-term stability of clinical and microbial improvements. Future randomized controlled trials should aim to address these issues through larger, multi-center cohorts, control comparisons, and longer-term follow-ups (e.g., 6–12 months).

In summary, SmartGel OV—a topical *Origanum-vulgare*-based gel—demonstrated clinically and microbiologically meaningful improvements when used as an adjunct in patients with periodontitis. Its anti-inflammatory, antimicrobial, and synergistic properties likely contributed to the observed outcomes. When combined with AI-driven inflammation tracking, the intervention also enabled early detection of non-responsiveness and supported patient engagement. This hybrid model of natural therapy and intelligent diagnostics offers a scalable, patient-centered, and minimally invasive approach to chronic periodontal care.

## 5. Conclusions

SmartGel OV, an *Origanum-vulgare*-based gel, showed promising adjunctive benefits for periodontitis, improving clinical outcomes and reducing key pathogens over 4 months. Its integration with an AI-based inflammation tracker supported non-invasive monitoring and patient engagement.

This dual approach combines natural anti-inflammatory therapy with intelligent diagnostics and presents a scalable model for personalized periodontal care.

While the findings are promising, further randomized controlled trials are essential to confirm these results. Moreover, future studies should focus on evaluating the long-term effectiveness and stability of SmartGel OV outcomes, particularly in diverse patient populations and across extended follow-up periods. Longitudinal data will be critical in validating the durability of clinical and microbial improvements and in establishing SmartGel OV as a reliable tool in integrative periodontal therapy. Future trials should also compare SmartGel OV with standard adjuncts (e.g., chlorhexidine and antibiotics) to determine its relative clinical value.

## Figures and Tables

**Table 1:** Mean Clinical Periodontal Parameters (*n* = 28).

Parameter	Baseline	4 Months
Probing Depth (PD, mm)	3.6 ± 0.5	2.2 ± 0.3
Clinical Attachment Level (CAL, mm)	4.8 ± 0.6	3.9 ± 0.5
Bleeding on Probing (BOP)	1.0 ± 0.2	0.2 ± 0.1
Plaque Index (PI)	1.1 ± 0.3	0.3 ± 0.2

**Table 2:** Prevalence of key periodontal pathogens before and after SmartGel OV use, showing notable reductions in red and orange complex species.

Pathogen	Baseline (%)	After 4 Months (%)
Aa	10.7	0.0
Pi	17.9	0.0
Pg	32.1	3.5
Tf	28.6	7.1
Td	25.0	3.5
Pm	21.4	3.5
Fn	42.9	10.7
Cr	14.3	3.5
En	10.7	0.0
Ec	7.1	0.0
Cp	35.7	7.1

## Data Availability

The data are not publicly available due to privacy or ethical restrictions.
